# Hyperimmune anti-COVID-19 IVIG (C-IVIG) Therapy for Passive Immunization of Severe and Critically Ill COVID-19 Patients: A structured summary of a study protocol for a randomised controlled trial

**DOI:** 10.1186/s13063-020-04839-5

**Published:** 2020-11-02

**Authors:** Shaukat Ali, Shobha Luxmi, Fatima Anjum, Sheikh Muhammad Muhaymin, Syed Muneeb Uddin, Ayesha Ali, Mir Rashid Ali, Sohaib Tauheed, Mujtaba Khan, Mohsin Bajwa, Saif Ullah Baig, Elisha Shalim, Iqra Ahmed, Abdul Samad Khan, Saeed Quraishy

**Affiliations:** 1 Dow College of Biotechnology, Dow University of Health Sciences, Karachi, Pakistan; 2 Dow University Hospital, Dow University of Health Sciences, Karachi, Pakistan; 3 Dow Research Institute of Biotechnology and Biomedical Sciences, Dow University of Health Sciences, Karachi, Pakistan; 4 Dow International Medical College, Dow University of Health Sciences, Karachi, Pakistan; 5 National Control Laboratory for Biologicals, Islamabad, Pakistan; 6 Dow University of Health Sciences, Karachi, Pakistan

**Keywords:** COVID-19, Randomised controlled trial, protocol, passive immunization, anti-COVID19 IVIG, single dose, C-IVIG, convalescent plasma

## Abstract

**Objectives:**

The aim of this trial is to investigate the safety and clinical efficacy of passive immunization therapy through Hyperimmune anti-COVID-19 Intravenous Immunoglobulin (C-IVIG: 5% liquid formulation), on severe and critically ill patients with COVID-19.

**Trial design:**

This is a phase I/II single centre, randomised controlled, single-blinded, superiority trial, through parallel-group design with sequential assignment. Participants will be randomised either to receive both C-IVIG and standard care or only standard care (4:1).

**Participants:**

The study is mono-centric with the participants including COVID19 infected individuals (positive SARS-CoV-2 PCR on nasopharyngeal and/or oropharyngeal swabs) admitted in institute affiliated with Dow University Hospital, Dow University of Health Sciences, Karachi, Pakistan. Consenting patients above 18 years that are classified by the treating physician as severely ill i.e. showing symptoms of COVID-19 pneumonia; dyspnea, respiratory rate ≥30/min, blood oxygen saturation ≤93%, PaO_2_/FiO_2_ <300, and lung infiltrates >50% on CXR; or critically ill i.e. respiratory failure, septic shock, and multiple organ dysfunction or failure. Patients with reported IgA deficiency, autoimmune disorder, thromboembolic disorder, and allergic reaction to immunoglobulin treatment were excluded from study. Similarly, pregnant females, patients requiring two or more inotropic agents to maintain blood pressure and patients with acute or chronic kidney injury/failure, were also excluded from the study.

**Intervention and comparator:**

The study consists of four interventions and one comparator arm. All participants receive standard hospital care which includes airway support, anti-viral medication, antibiotics, fluid resuscitation, hemodynamic support, steroids, painkillers, and anti-pyretics. Randomised test patients will receive single dose of C-IVIG in following four dosage groups:

Group 1: 0.15g/Kg with standard hospital care

Group 2: 0.2g/Kg with standard hospital care

Group 3: 0.25g/Kg with standard hospital care

Group 4: 0.3g/Kg with standard hospital care

Group 5 (comparator) will receive standard hospital care only

**Main outcomes:**

The primary outcomes are assessment and follow-up of participants to observe 28-day mortality and,

• the level and duration of assisted ventilation during hospital stay,

• number of days to step down (shifting from ICU to isolation ward),

• number of days to hospital discharge,

• adverse events (Kidney failure, hypersensitivity with cutaneous or hemodynamic manifestations, aseptic meningitis, hemolytic anemia, leuko-neutropenia, transfusion related acute lung injury (TRALI)) during hospital stay,

• change in C-Reactive Protein (CRP) levels,

• change in neutrophil lymphocyte ratio to monitor inflammation.

**Randomisation:**

Consenting participants who fulfill the criteria are allocated to either intervention or comparator arm with a ratio of 4:1, using sequentially numbered opaque sealed envelope simple randomization method. The participant allocated for intervention will be sequentially assigned dosage group 1-4 in ascending order. Participants will not be recruited in the next dosage group before a set number of participants in one group (10) are achieved.

**Blinding (masking):**

Single blinded study, with participants blinded to allocation.

**Numbers to be randomised (sample size):**

Total 50 patients are randomised. The intervention arms consist of 40 participants divided in four groups of 10 participants while the comparator group consists of 10 patients.

**Trial Status:**

Current version of the protocol is “Version 2” dated 29^th^ September, 2020.

Participants are being recruited. Recruitment started on June, 2020 and is estimated to primarily end on January, 2021.

**Trial registration:**

This trial was registered at ClinicalTrials.gov, NCT04521309 on 20 August 2020 and is retrospectively registered.

**Full protocol:**

The full protocol is attached as an additional file, accessible from the Trials website (Additional file 1).

**Supplementary Information:**

**Supplementary information** accompanies this paper at 10.1186/s13063-020-04839-5.

## Supplementary Information

**Additional file 1.** Full Study Protocol.

## Data Availability

Not applicable.

