# Supplementary material for: Hyperimmune anti-COVID-19 IVIG (C-IVIG) Therapy for Passive Immunization of Severe and Critically Ill COVID-19 Patients: A structured summary of a study protocol for a randomised controlled trial
Source: Trials. 2020 Nov 2;21:905. doi: 10.1186/s13063-020-04839-5 (PMC7604645; doi:10.1186/s13063-020-04839-5)
Supplement: Supplementary file 1 — Additional file 1. Full Study Protocol. [file 13063_2020_4839_MOESM1_ESM.pdf]

SARS-CoV-2 Antibodies based IVIG (C-IVIG) Therapy of Severe and Critically Ill COVID-19 Patients

Study Protocol
NCT04521309

29th September, 2020

Objective

The aim of this trial is to investigate the safety and clinical efficacy of passive immunization therapy through Hyperimmune anti-COVID-19 Intravenous Immunoglobulin (C-IVIG: 5% liquid formulation), on severe and critically ill COVID-19 patients.

Methodology

Plasma collection from donors (recovered COVID-19 individuals):

1. Selection of donor according to World Health Organization (WHO) and Federal Drug Agency (FDA) guidelines

Inclusion Criteria for Donor	*Exclusion criteria for Donor*
a. Submitted signed consent<br>b. Eligible to donate blood<br>c. Negative for HIV, HBV, HCV, syphilis and malarial parasite<br>d. Prior diagnosis of COVID-19 documented by a laboratory test<br>e. Complete resolution of symptoms at least 14 days prior to donation<br>f. Female donors negative for HLAantibodies or male donors<br>g. Negative results for COVID-19 either from one or more nasopharyngeal swab specimens or by a molecular diagnostic test from blood.<br>h. Defined SARS-CoV-2 neutralizing antibody titers, if testing can be conducted (e.g., optimally greater than 1:320	a. Pre-existing condition Contra indicative for donating blood (HIV, viral hepatitis, tuberculosis, syphilis, oncological conditions, malaria)<br>b. Bleeding tendency<br>c. Anemia<br>d. Fever of unknown origin

2. Blood collection and plasma separation

300-1000 ml plasma will be collected from consenting COVID-19 recovered patients (two weeks after recovery and having evidence of negative PCR for SARS CoV-2) using plasmapheresis technique. Plasmapheresis is a technique where using a machine the blood components are separated, keeping the required component, in this case plasma, and returning other components like RBCs.Plasma will be stored according to WHO guidelines. Aliquot of plasma will be subjected to ABO blood typing and screened for syphilis, malarial parasite HIV, HBV, HCV, and COVID – 19 by Nucleic Acid Test (NAT). The screened stored plasma qualifying safety criteria will be pooled and fractionated to obtain Anti-COVID hyperimmune immunoglobulin.

Administration of extracted IgG in respective doses by medical Experts and researchers in selected recipient

Eligibility Criteria for Recipient:

Revised Inclusion criteria for study participant (Covid-19 patients)	***Exclusion criteria for Recipient***
1. Above 18 years of age<br>2. Have positive COVID PCR on nasopharngeal and/or oropharyngeal swabs<br>3. Admitted in isolation ward and ICU of institutes affiliated with DUHS<br>4. have severe or life threatening COVID as judged by the treating physician<br>5. Consent given by the patient or first degree relative<br><br>Severe COVID-19 is defined by one or more of the following:<br>dyspnea<br>respiratory frequency ≥ 30/min<br>blood oxygen saturation ≤ 93%<br>partial pressure of arterial oxygen to fraction of inspired oxygen ratio < 300<br>lung infiltrates > 50% within 24 to 48 hours<br>Life-threatening COVID-19 is defined as one or more of the following:<br>respiratory failure<br>septic shock<br>multiple organ dysfunction or failure	a. Pregnancy<br>b. Previous allergic reaction to immunoglobulin treatment<br>c. Ig A deficiency<br>d. Patient requiring 2 inotropic agents to maintain blood pressures<br>e. Known case of any autoimmune disorder<br>f. Acute kidney injury or chronic renal failure<br>g. Known case of thromboembolic disorder<br>h. Aseptic meningitis

Study Design

This is a phase I/II single centred, randomized controlled, single-blinded, superiority trial, through parallel-group design with sequential assignment. Participants will be randomized either to receive both C-IVIG and standard care or only standard care (4:1). The study consists of intervention comprising of four arms with each study arm containing 10 participants. All participants receive standard hospital care which includes airway support, anti-viral medication, antibiotics, fluid resuscitation, hemodynamic support, steroids, painkillers, and anti-pyretic. Randomized test patients will receive single dose of C-IVIG in following four dosage groups:
Group 1: 0.15g/Kg with standard hospital care
Group 2: 0.2g/Kg with standard hospital care
Group 3: 0.25g/Kg with standard hospital care
Group 4: 0.3g/Kg with standard hospital care

Group 5 (comparator) will receive standard hospital care only.
The primary outcomes are assessment and follow-up of participants for 28 days to check for mortality and,
- the level and duration of assisted ventilation during hospital stay,

- number of days to step down (shifting from ICU to isolation ward),
- number of days to hospital discharge,
- adverse events (Kidney failure, hypersensitivity with cutaneous or hemodynamic manifestations, aseptic meningitis, hemolytic anemia, leuko-neutropenia, transfusion related acute lung injury (TRALI)) during hospital stay,
- change in C-Reactive Protein (CRP) levels,
- change in neutrophil lymphocyte ratio to monitor inflammation.
